# Diversity-oriented synthesis derived indole based spiro and fused small molecules kills artemisinin-resistant *Plasmodium falciparum*

**DOI:** 10.1186/s12936-021-03632-2

**Published:** 2021-02-17

**Authors:** Akshaykumar Nayak, Himani Saxena, Chandramohan Bathula, Tarkeshwar Kumar, Souvik Bhattacharjee, Subhabrata Sen, Ashish Gupta

**Affiliations:** 1 Epigenetics & Human Disease Laboratory, Department of Life Sciences, Shiv Nadar University, Uttar Pradesh, NH-91, Tehsil-Dadri, Greater Noida, 201314 India; 2 Department of Chemistry, Shiv Nadar University, Uttar Pradesh, Tehsil-Dadri, Greater Noida, 201314 India; 3 Special Centre for Molecular Medicine, Jawaharlal Nehru University, New Delhi, India

**Keywords:** *Plasmodium*, Artemisinin, Artemisinin-resistance, DOS, Indole

## Abstract

**Background:**

Despite numerous efforts to eradicate the disease, malaria continues to remain one of the most dangerous infectious diseases plaguing the world. In the absence of any effective vaccines and with emerging drug resistance in the parasite against the majority of anti-malarial drugs, the search for new drugs is urgently needed for effective malaria treatment.

**Methods:**

The goal of the present study was to examine the compound library, based on indoles generated through diversity-oriented synthesis belonging to four different architecture, i.e., 1-aryltetrahydro/dihydro-β-carbolines and piperidine/pyrrolidine-fused indole derivatives, for their in vitro anti-plasmodial activity. Trifluoroacetic acid catalyzed transformation involving tryptamine and various aldehydes/ketones provided the library.

**Results:**

Among all the compounds screened, 1-aryltetrahydro-β-carbolines 2 and 3 displayed significant anti-plasmodial activity against both the artemisinin-sensitive and artemisinin-resistant strain of *Plasmodium falciparum*. It was observed that these compounds inhibited the overall parasite growth in intra-erythrocytic developmental cycle (IDC) via reactive oxygen species-mediated parasitic death and thus could be potential anti-malarial compounds.

**Conclusion:**

Overall the compounds 2 and 3 identified in this study shows promising anti-plasmodial activity that can kill both artemisinin-sensitive and artemisinin-resistant strains of *P. falciparum*.

## Background

Since primitive times, malaria, a mosquito-borne infectious disease, has remained the leading cause of mortality from any parasitic disease around the world. It has been estimated that *Plasmodium* spp., the causative agent of malaria, infected 228 million people, accounting for about half a million deaths in 2019, mostly affecting poor people living in tropical and sub-tropical regions of the world [1]. Unlike other *Plasmodium* species, *Plasmodium falciparum* causes the severe form of malaria and poses higher risk of death due to associated neurological, renal or cardiological complications [2]. To reduce the number of malaria-related cases and mortality, most of the malaria control programmes besides using other control measures, rely heavily on killing the malarial parasite by anti-malarial chemotherapy [3]. Quinine, a plant derived chemical extracted from the bark of the Cinchona tree, was used to treat malaria from as early as the 1600 s and is still used as second-line therapy for the management of uncomplicated malaria in Africa when the first-line drug either fails or is not available [4–7]. With the range of adverse side effects experienced by the use of quinine, the search for a safe and most effective anti-malarial medicine led to the discovery of chloroquine, followed by many synthetic anti-malarials, e.g., sulfadoxine-pyrimethamine, amodiaquine and mefloquine [5, 6, 8, 9]. Despite their early success in therapeutic management of malaria, their overuse contributed to the development and spread of resistance against these drugs by the malarial parasite. Undoubtedly, the unregulated use of these drugs as monotherapy further accelerated their failing therapeutic efficacy [6, 9–11].

At present, the most potent and successful drug available for the treatment of both severe and uncomplicated malaria is artemisinin, which was derived from the Qinghao plant (*Artemisia annua*) in the 1970s [5]. Artemisinin is frequently used in combination with a partner anti-malarial drug to overcome its pharmacokinetic limitations (such as poor bioavailability, low solubility in water and a relatively shorter half-life in vivo (~ 2.5 h)) and to protect its efficacy against parasite resistance for a longer period of time [5]. Presently, five recommended artemisinin-based combinations include combinations of artemisinin derivates, such as artemisinin, dihydroartemisinin, artemether, artesunate, with lumefantrine, mefloquine, amodiaquine, sulfadoxine/pyrimethamine, piperaquine, and chlorproguanil/dapsone [5]. Artemisinins are particularly active against the ring and mature trophozoite form of asexual life-cycle stage of parasites persisting within infected red blood cells. However, a sub-population of ring form of the parasite may tolerate artemisinin by becoming temporarily dormant or sequestered to return after a few days or weeks, eventually causing the failure of treatment [6, 12]. It is for this reason that artemisinin monotherapy is not preferred and is recommended to be used only with longer-acting partner anti-malarial drug that would kill the surviving dormant form of the parasite [11, 13]. Although artemisinin-based combination therapy (ACT) had a marked effect on malaria cases globally, the appearance of artemisinin-resistant cases in Southeast Asia, especially in the eastern Greater Mekong Sub-region, is cause for concern [14–17]. By relying so heavily upon the use of ACT, eventually this valuable anti-malarial drug will become ineffective, given the history of resistance development in the parasite to most anti-malarials. Bringing safe and new anti-malarial drug candidates with diverse chemical structures and mechanism of action into clinical trials is critical to combat emerging anti-malarial drug resistance in the parasite.

Over time, new concepts in organic synthesis and molecular design, such as fragment-based drug discovery (FBDD), ligand-based drug discovery (LBDD), biology-oriented synthesis (BIOS), and diversity-oriented synthesis (DOS) began to evolve. Compared to traditional drug discovery platforms, these methods have not only expedited the process of bringing new drugs onto the market but also have helped in providing drugs with better specificity towards the target and lesser toxicity. DOS has particularly emerged as a synthetic approach that is used for the design and construction of novel, small molecule libraries containing a high degree of structural and stereo-chemical diversity [18, 19]. Screening of DOS-derived compound libraries has led to identification of many novel and biologically useful small molecules known for their antibacterial, antifungal, antiparasitic, and anticancer properties [20–25]. Accordingly, the present work aimed to screen and evaluate compounds of DOS library (comprised of 11 indole-based heterocycles which are connected to piperidine or pyrrolidine molecules either through bond fusion or via spiro linkage) for their efficacy in killing both wild-type and artemisinin-resistant strains of *P. falciparum* in vitro. These compounds can be segregated into 4 different structures. The initial reaction involved trifluoroacetic acid (TFA) catalyzed condensation reactions of tryptamines with various aldehydes/ ketones to afford various library molecules (Experimental procedure section, Additional file 1). Few of them were further transformed to newer compounds by *N*-bromosuccinimide (NBS)-mediated oxidation or ring contraction (Experimental procedure section, Additional file 1).

The study’s screening assay identified that 1-aryltetrahyro-β-carboline class of compounds possess significant anti-plasmodial activity. Further screening of two best compounds, i.e., 2 and 3, showed their potential to kill both wild-type and artemisinin-resistant strains. Also shown was that compound 3 can induce significant reactive oxygen species (ROS) generation in malaria parasites, providing insight into the mechanism of compound 3-induced parasite death.

## Methods

### Compound designs and synthesis

The DOS library screened here is comprised of 11 compounds belonging to four different structural classes of compounds, as described in Additional file 1 and previously published work [26, 27]. They are dihydro and tetrahydro-β-carbolines, piperidine and pyrrolidine-fused tetrahyro-β-carbolines and spiropyrrolooxoindoles (Additional file 1: Figure S1A). Tryptamine is the appropriate substrate which when reacted with aldehydes/ketones in presence of catalytic TFA, undergoes condensation to provide a preliminary set of scaffolds 1→3, 9 and 11 (Additional file 1: Figure S1B). Compound 2 and a few more tetrahydro-β-carbolines 5a-8a were oxidized to dihydro-β-carbolines 4–8 in presence of NBS (*N*-Bromosuccinimide) and compound 9 underwent NBS-mediated ring contraction to afford a diastereomeric mixtures of 10a/b (Additional file 1: Figure S1B). Detailed synthesis and characterization of compounds is provided in Additional file 1.

### Parasite culture

*Plasmodium falciparum* artemisinin-sensitive parental 3D7 strain and artemisinin-resistant R539T strain (expressing PfKelch13/PF3D7_1343700 with R539T mutation) were cultured in O+ ve erythrocytes at 37 °C. The generation and characterization of artemisinin-resistant parasites were reported earlier [28, 29]. RPMI-1640 media (supplemented with 0.5% albumax, 0.2% sodium bicarbonate, 50 µg/ml of hypoxanthine, and 10 µg/ml of gentamicin) and mixture gas environment (90% nitrogen, 5% oxygen and 5% carbon dioxide) was used for culture. For routine culture, haematocrit was maintained at 5% and the stages and growth of parasite was routinely monitored by microscopic examination of Giemsa-stained blood smears. Synchronization of parasite culture was done using 5% sorbitol method as discussed elsewhere [30].

### Parasite growth inhibition assay

To investigate the inhibitory potential of compounds, parasite growth inhibition assay was performed with parasite culture synchronized in ring stage and seeded in 12-well plates at ~ 1% parasitaemia. These parasites were treated with compounds at various concentrations for 48 h. Microscopy-based examination of the Giemsa-stained blood smears of parasite culture treated either with compounds or DMSO (control) were performed at 0 and 48 h and parasites in different stages of intra-erythrocytic developmental cycle (IDC) were counted. For the calculation of IC_50_ values, GraphPad Prism 5.0 software was used.

### Measurement of ROS levels

The detection of ROS level in parasitized-erythrocyte was performed using H_2_DCFDA dye. For this, parasite culture was treated with compounds for 48 h and then washed with 1X PBS. Treated parasites were incubated with H_2_DCFDA dye (5 µM) and incubated for 20 min in dark and subsequently washed using 1X PBS. Thin blood smears were prepared on glass slides and mounted using anti-fade DAPI. ROS generation was detected under confocal microscope (Nikon Eclipse Ti2-E) using 100× magnification. Intensity of generated signal was calculated using NIS-Elements AR analysis 5.20.02 software.

## Results

### Evaluation of DOS-derived compounds for their anti-plasmodial activity

**Fig. 1:**
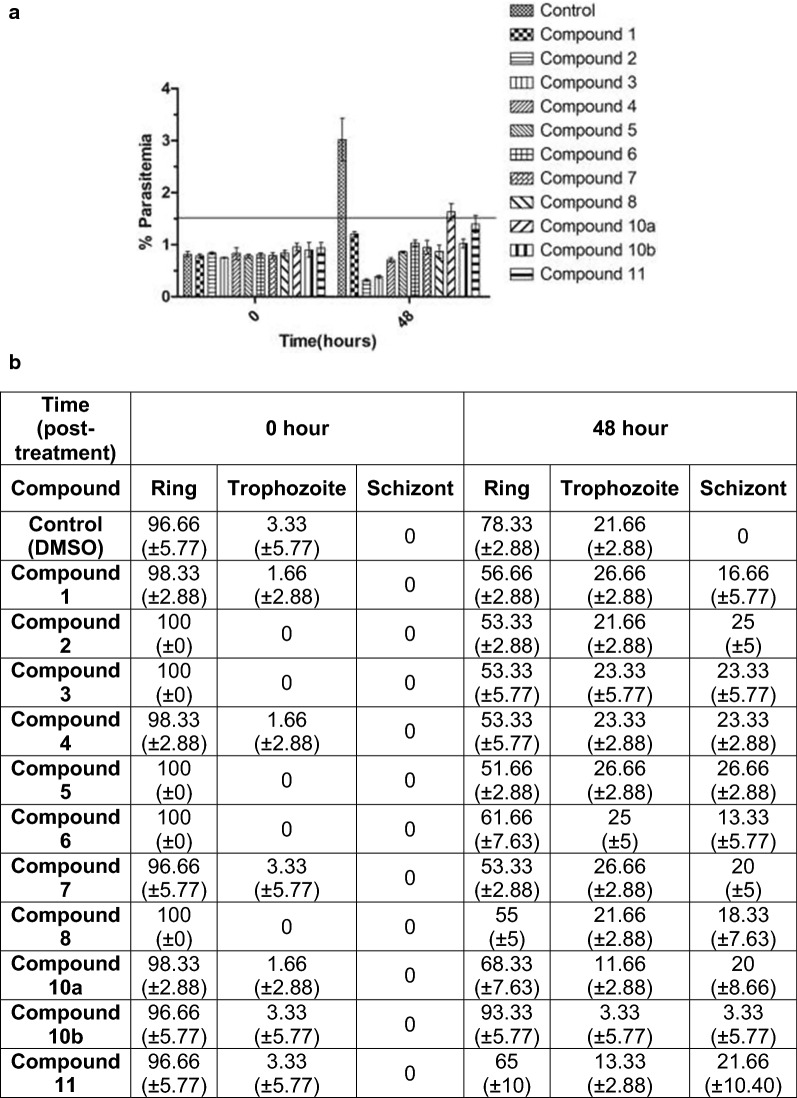
Screening of DOS-synthesised compounds for anti-plasmodial activity. **a** Synchronized parasite culture in ring stage were seeded to adjust parasitaemia to 1%. This synchronized culture was then treated either with DMSO or compounds (5 µM) for 48 h. Giemsa-stained slides were prepared at 0 and 48 h post-treatment to monitor the parasitaemia. Graphs were prepared for the average value of three independent experiments performed in duplicate with ± SD. **b** Parasites at different stages of the IDC at 0 and 48 h post-treatment were calculated. Table shows the percentage of remaining parasites in different stages of IDC from three independent experiments with ± SD

The DOS-derived compound library (Additional file 1: Figure S1) was screened for anti-plasmodial activity using parasite growth inhibition assay. For this, synchronized ring-stage parasite culture of 3D7 strain was treated with the compounds at 5 μM concentration for 48 h. Parasites treated with DMSO were used as control. Parasitaemia was measured at 0 and 48 h post-treatment and graph was plotted for the average value of three independent sets of experiments (Fig. 1a). Fifty percent parasite growth inhibition was reserved as cut-off value for selection of compounds to have anti-plasmodial activity. Results showed that 1-aryltetrahydro-β-carbolines 1–3 and 1-aryldihydro-β-carbolines 4–8 were most efficacious, demonstrating more than 50% inhibition of *Plasmodium* growth (Fig. 1a).

Intra-erythrocytic developmental cycle (IDC) is an asexual replicative phase characterized by the parasite progression through three successive morphological stages as ring, trophozoite and schizont. Next, the effect of 1-aryl-dihydro/tetrahydro-β-carbolines 1–8 on growth progression of *Plasmodium* during the IDC in presence of these compounds was evaluated. Results showed that compounds 1–8 killed all the IDC stages of the parasite as no stage-specific growth inhibition was observed (Fig. 1b). Figure 1b shows the percentage of parasite population in different stages of IDC in control and treated samples at 0 and 48 h post-treatment. Together the phenotypic screening identified 8 compounds effective in killing *P. falciparum* parasites with their toxicity independent of the parasite stages.

### Compounds 2 and 3 inhibit artemisinin-resistant parasite growth

**Fig. 2:**
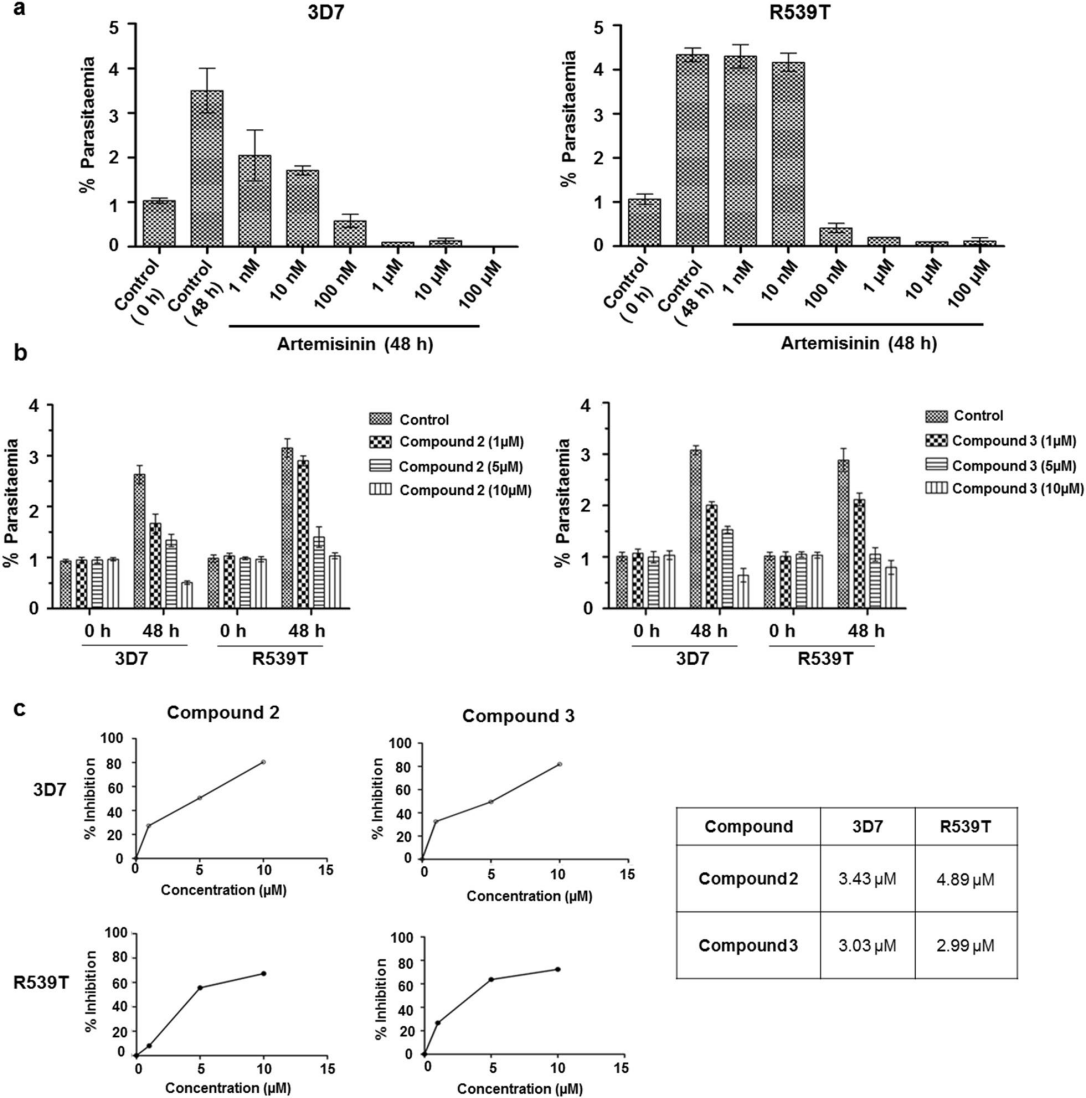
Compounds 2 and 3 inhibit artemisinin-resistant parasite growth. **a** Synchronized ring-stage culture of artemisinin-sensitive (3D7) and artemisinin-resistant (R539T) parasites at 1% parasitaemia were treated with DMSO or different concentrations of artemisinin for 48 h. Giemsa-stained slides of treated culture were analysed to determine the parasitaemia at 0 and 48 h post-treatment. **b** Ring-stage synchronized culture of *P. falciparum* 3D7 and R539T strains were treated either with DMSO or compounds 2 or 3 at different concentrations for 48 h. Parasitaemia was calculated by examining Giemsa-stained slides prepared at 0 and 48 h post-treatment. **c** Graphs were plotted for percentage inhibition in parasitaemia at 48 h for different concentration and IC_50_ value was calculated for both compounds in artemisinin-sensitive (3D7) and artemisinin-resistant strains (R539T)

After observing significant inhibitory effect of β-carboline derivatives on wild-type 3D7 strain growth and survival, the interest was to determine the inhibitory effect of the most potent β-carboline derivatives 2 and 3 on artemisinin-resistant *P. falciparum* (R539T) strain. Primarily to determine the artemisinin-resistance of R539T strain, synchronized ring-stage parasites of 3D7 and R539T parasite strains were treated with different concentrations of artemisinin (1 nM, 10 nM, 100 nM, 1 µM, 10 µM, 100 µM) or DMSO (control) for 48 h. Results showed significant resistance to artemisinin in R539T parasite (Fig. 2a). IC_50_ value of artemisinin was calculated to be 3.86 nM and 40.3 nM for artemisinin-sensitive 3D7 and artemisinin-resistant R539T parasites, respectively. Next, to determine the effect of selected compounds (β-carboline derivatives 2 and 3) on artemisinin-resistant parasite, synchronized ring-stage parasites of R539T strain were incubated with different concentrations (1 µM, 5 µM, 10 µM) of these two compounds or DMSO (control) for 48 h. The parental 3D7 parasites were also treated simultaneously in similar manner for comparative analysis. Results showed corresponding reduction in parasitaemia with increasing concentration of these compounds in both artemisinin-sensitive (3D7) and artemisinin-resistant (R539T) strains (Fig. 2b). The IC_50_ value of compound 2 was calculated to be 3.43 ± 0.77 µM and 4.89 ± 0.91 µM for 3D7 and R539T strains, respectively, while for compound 3 it was 3.03 ± 0.24 µM and 2.99 ± 0.48 µM for 3D7 and R539T strains, respectively (Fig. 2c). Together these findings showed that compounds 2 and 3 can potently inhibit the growth and survival of both wild-type and artemisinin-resistant parasites.

Since compounds 2 and 3 showed promising anti-plasmodial activity, the SwissADME web tool [31] was used to determine their suitability as potential drug candidates through analysing some of the key ADME parameters, such as solubility and lipophilicity. The aqueous solubility (Log S[ESOL]) for compounds 2 and 3 was calculated to be -5.65 and -4.01, respectively, and show that these compounds are fairly soluble in water. Similarly, lipophilicity (log P_o/w_) for compounds 2 and 3 was calculated to be 3.09 and 3, respectively, which are within the acceptable ADME parameters.

### Compounds 2 and 3 enhance ROS generation in *Plasmodium falciparum*

**Fig. 3:**
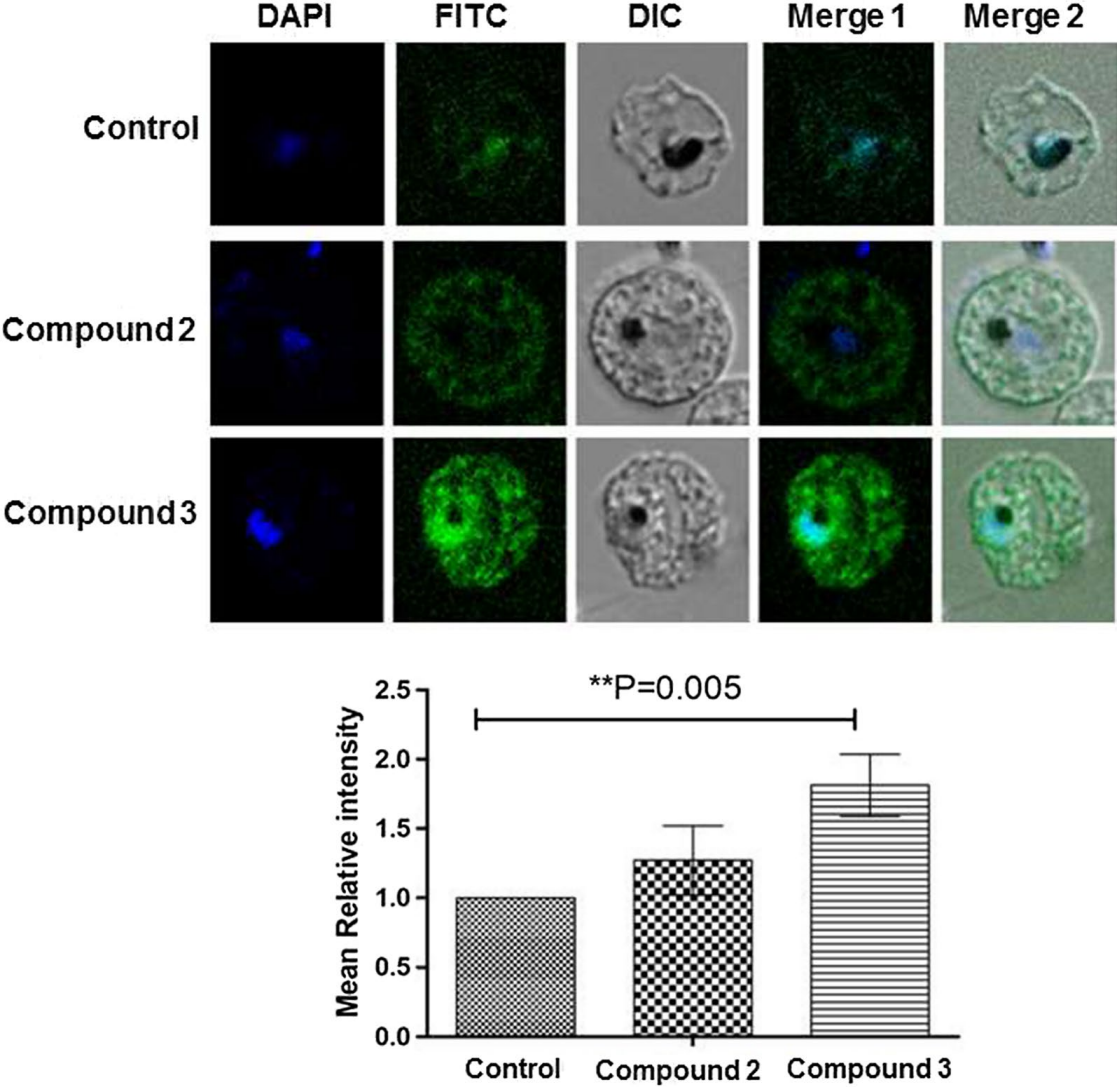
Compounds 2 and 3 enhance ROS generation in *Plasmodium*. Parasites were treated with DMSO or compounds 2 or 3 for 48 h followed by H_2_DCFDA dye staining. Signal was detected using fluorescence microscope. DAPI was used to stain the parasite nucleus. Graph was plotted for relative intensity of H_2_DCFDA dye signal. P value (0.005) shows significant enhancement in ROS generation

To determine whether the growth inhibitory effect of β-carbolines compounds 2 and 3 was associated with generation of oxidative stress, the level of ROS in the presence of these compounds was examined by the H_2_DCFDA dye staining method. For this, *Plasmodium* infected red blood cells (RBCs) were treated with compounds 2 and 3 for 48 h followed by staining with H_2_DCFDA dye. ROS generation was detected using fluorescence microscopy and the result showed significant enhancement in the level of ROS generation with compound 3 compared to control (Fig. 3). Compound 2-treated parasites also showed ROS generation, however at lower levels compared to compound 3.

## Discussion

Despite availability of a wide variety of anti-malarial drugs, including quinine, chloroquine, sulfadoxine, atovaquone, primaquine, artemisinin, and their derivatives, increasing cases of drug resistance in malaria parasites and transmission of resistant parasites in new areas is throwing a challenge for worldwide efforts to eradicate malaria [32, 33]. There is a need to come up with new anti-malarials with different mechanisms of action. DOS method has led to generation of libraries of small molecules based on complex structure scaffolds and has successfully played a role in the discovery of several bioactive compounds that have either emerged as drug leads or have helped enhance the knowledge of complex biological processes [34–36].

The presence of indole-based heterocycles in many bioactive natural products has prompted scientists to use indole scaffold for developing libraries of a diverse range of compounds that are known to show promising activity against many human diseases, including cancer, microbial infection and parasitic diseases [37, 38]. This has encouraged synthesis of libraries comprising of molecules inspired from various indole heterocycles and natural products as potential lead candidates against malaria parasites [39–42]. In this study, a library of indole- based spiro and fused small molecules were screened and among all the compounds tested, compounds 2 and 3 exhibited toxicity against all the parasitic stages of IDC with similar levels of lethality for artemisinin-sensitive and artemisinin-resistant strains of *P. falciparum.* This suggest that these compounds might have a dissimilar mode of action compared to artemisinin for killing malaria parasite. The compounds showed strong anti-plasmodial activity at a concentration range of 3–5 µM which is in comparable range to other indole-based anti-plasmodial compounds shown in previous studies [40, 41].

Compound 3 induced oxidative stress by enhancing production of ROS, which could be a possible mechanism of action of this compound in killing malaria parasites. ROS are known to damage DNA, RNA and can oxidize proteins and lipids, leading to death of a cell. Bioactive indole compounds are shown to bind with multiple receptors, and thus can act by multiple mechanisms making them attractive chemical molecules for developing novel therapeutic compounds [38]. Hit compounds identified in this study can be further refined and developed for use as anti-malarial compounds or can be used as a scaffold to develop artemisinin-based hybrid molecules [43].

## Conclusions

Overall, this study displays the potential of 1-aryltetrahydro-β-carbolines compounds as anti-malarials that can kill artemisinin-resistant parasites. This opens the possibility to develop them or their derivatives as combination drugs that can be used with existing anti-malarials.

## Supplementary Information

**Additional file 1.** Supplementary Material.

## Data Availability

All data generated or analysed during this study are included in this published article [and its additional information files].
