# Supplementary material for: Diversity-oriented synthesis derived indole based spiro and fused small molecules kills artemisinin-resistant Plasmodium falciparum
Source: Malar J. 2021 Feb 17;20:100. doi: 10.1186/s12936-021-03632-2 (PMC7891021; doi:10.1186/s12936-021-03632-2)

Diversity-oriented synthesis derived indole based spiro and fused small molecules kills artemisinin-resistant *Plasmodium falciparum*

Akshaykumar Nayak^1#^, Himani Saxena^1#^, Chandramohan Bathula^2^, Tarkeshwar Kumar^3^, Souvik Bhattacharjee^3^, Subhabrata Sen^2^*, Ashish Gupta^1^*

**Fig. S1**

**A**


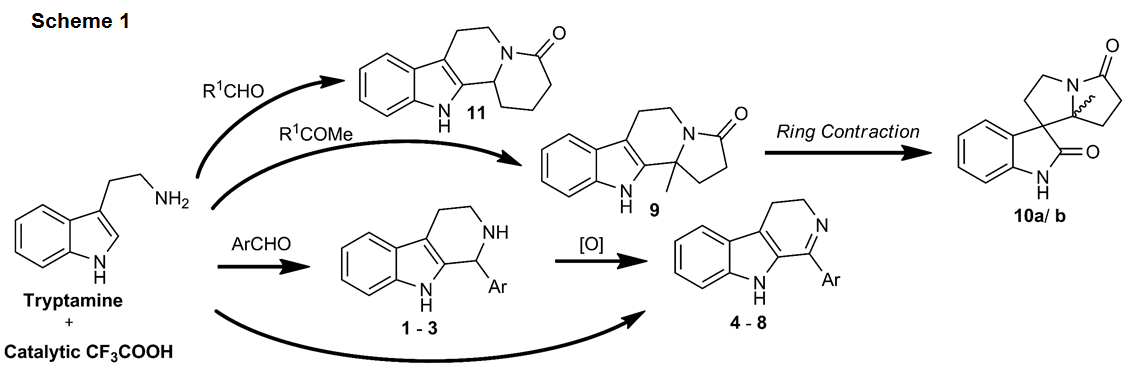


**B**


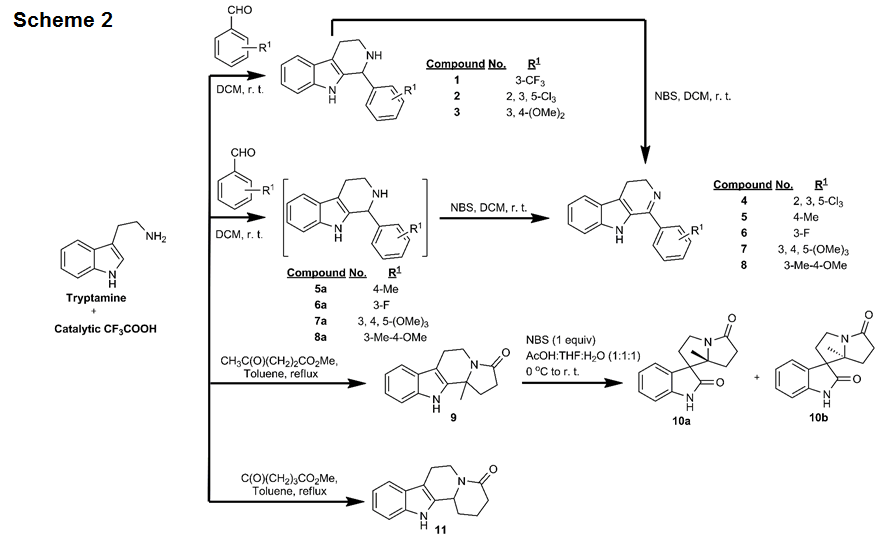


**Experimental procedure**

**Synthesis of compounds 1** → **11**

The synthesis of the molecular library began by the condensation of tryptamine with appropriate aromatic and aliphatic aldehyde/ketones in presence of catalytic TFA. The temperature of the reactions ranged from 25 to 80°C (Scheme 1). Compounds **1**-**3** were synthesized by the reactions of tryptamine with 3-triflurobenzaldehyde, 2, 3, 5-trichlorobenzaldehyde and 3, 4-dimethoxybenzaldehyde respectively (Scheme 1). Next, compound **2** was oxidized by N-bromosuccinimide (NBS) to afford the corresponding dihydro-β-carboline **4**. In a similar manner the series of 1-aryl-dihydro-β-carbolines **5**-**8** were synthesized from tryptamine and corresponding aryl aldehydes to generate the 1-aryl-tetrahydro-β-carboline **4a**-**8a**, as crude intermediates which were then oxidized by NBS to provide the desired compounds (Scheme 1). Refluxing tryptamine in catalytic TFA with methyl levulinate afforded compound **9**. Compound **9** when treated with NBS in presence of 1:1:1 water (H_2_O): tetrahydrofuran (THF): acetic acid (AcOH) from 0°C to r. t., underwent ring contraction to afford a pair of diastereomeric spiropyrrolo oxoindole **10a**/**b** in 1:1 ratio (Scheme 1). Finally refluxing 1-oxo-5-methylpentanoate with tryptamine in catalytic TFA provided compound **11** (Scheme 1).

**General experimental plan**

All reactions were carried out in flame-dried sealed tubes with magnetic stirring. Unless otherwise noted, all experiments were performed under argon atmosphere. All reagents were purchased from Sigma Aldrich, Acros or Alfa Aesar. Solvents were treated with 4 Å molecular sieves or sodium and distilled prior to use. Purifications of reaction products were carried out by column chromatography using Chem Lab silica gel (230-400 mesh). Infrared spectra (IR) were recorded on a Thermoscientific Neoled IS5 FTIR spectrophotometer and are reported as wavelength numbers (cm^-1^). Infrared spectra were recorded by preparing a KBr pellet containing the title compound. ^1^H NMR and ^13^C NMR spectra were recorded with tetramethylsilane (TMS) as internal standard at ambient temperature unless otherwise indicated on a Varian 300/400 and JEOL JNM-ECX500 MHz at 500 MHz for ^1^H NMR and 100 MHz for ^13^C NMR. Chemical shifts are reported in parts per million (ppm) and coupling constants are reported as Hertz (Hz). Splitting patterns are designated as singlet (s), broad singlet (bs), doublet (d), triplet (t). Splitting patterns that could not be interpreted or easily visualized are designated as multiple (m). Mass spectrometry analysis was done on the 6540 UHD Accurate-Mass Q-TOF LC/MS system (Agilent Technologies) equipped with Agilent 1290 LC system obtained by the Dept. of Chemistry, School of Natural Sciences, Shiv Nadar University, Uttar Pradesh 201314, India. Compounds **1**, **2** and **3** are known compounds and their data are also available in the literature (Reference 26 and 27).

**General experimental procedure for the synthesis of**

**3, 4-dihydro-β-carbolines**

Tryptamine (160 mg, 1 mmol) and appropriate aldehyde (1 mmol) was dissolved in 10 mL of dichloromethane and was stirred in presence of trifluoroacetic acid (10 mol%) at r. t. Once TLC indicates complete consumption of the starting material, the solution was filtered through celite and the solvent evaporated to obtain the crude tetrahydro-β-carbolines **2, 5a**-**8a**, which were used as such in the next reaction.

To a solution of the appropriate tetrahydro-β-carbolines (1 eq.) in 10 mL of toluene was added N-bromosuccinimide (NBS) (1.1 eq.) and the resulting solution was stirred at 0°C for 6 hrs after which it was gradually warmed to room temperature (rt). Once TLC confirms the total consumption of the starting imine, the reaction was quenched with water. The aqueous layer was extracted twice with ethyl acetate. The organic extracts were washed with brine, dried over anhydrous magnesium sulphate and was evaporated to provide the crude compound. It was purified by column chromatography using 20% ethyl acetate in hexane as eluent to afford the desired 3, 4 –dihydro-β-carbolines **4**-**8**.

**1-(2, 3, 5-trichlorophenyl)-4,9-dihydro-3H-pyrido[3,4-b]indole (4)**

Following the general procedure crude **2** (321 mg) `1 (which was synthesized *via* general protocol from tryptamine and 2,3,5-trichlorobenzaldehyde (209 mg, 1 mmol)), with NBS (196 mg, 1.1 mmol) in toluene (10 mL) provided the desired compound **4** in 226 mg (yield 65%) as a white solid.^1^H NMR (500 MHz; DMSO-d_6_): 11.05 (s, 1H); 8.01-8.00 (d, *J*= 2.05 Hz, 1H); 7.62-7.57 (m, 2H); 7.34-7.32 (d, *J*= 8.25 Hz, 1H); 7.21-7.18 (t,*J_1_* = 14.4 Hz, *J*_2_ = 6.85 Hz, 1H); 7.08-7.05 (t,*J_1_* = 14.4 Hz, *J_2_* = 6.85 Hz, 1H); 3.98-3.94 (t,*J_1_* = 17.2 Hz, *J_2_* = 8.25 Hz, 2H); 2.94-2.90 (t,*J_1_* = 17.15 Hz, *J_2_* = 8.9 Hz, 2H). ^13^C NMR (125 MHz; DMSO-d_6_): 156.85, 140.18, 136.97, 133.97, 132.44, 130.33, 129.23, 128.91, 127.55, 124.75, 124.04, 119.78, 119.63, 115.20, 112.47, 48.75, 18.75. HRMS (ESI-TOF) m/z: [M + H]^+^Calcd for C_17_H_12_Cl_3_N_2_349.0061 Found 349.0091; IR 3063.65, 2830.83, 1738.211537.81.

**1-(p-tolyl)-4, 9-dihydro-3H-pyrido[3,4-b]indole (5)**

Following the general procedure crude **5a** (234 mg) (which was synthesized *via* general protocol from tryptamine and 4-methylbenzaldehyde (120 mg, 1 mmol)), with NBS (196 mg, 1.1 mmol) in toluene (10 mL) provided the desired compound **5** in 161 mg (yield 62%) as a white solid. ^1^H NMR (500 MHZ; DMSO-d_6_): 11.12 (s, 1H); 7.67-7.66 (d, *J* = 8.25 Hz, 2H); 7.62-7.60 (d, *J* = 7.55 Hz, 1H); 7.44-7.42 (d, *J* = 8.2 Hz, 1H); 7.34-7.32 (d, *J* = 8.25 Hz, 2H); 7.22- 7.19 (m, 1H); 7.09- 7.06 (t, *J_1_* = 14.45 Hz, *J_2_* = 7.55 Hz,1H); 3.88- 3.85 (t, *J_1_* = 16.45 Hz, *J_2_* = 8.2 Hz, 2H); 2.87- 2.84 (t, *J_1_* = 16.5 Hz, *J_2_* = 8.25 Hz,2H); 2.39 (s, 3H). ^13^C NMR (125 MHz; DMSO-d_6_): 158.49, 139.33, 136.93, 134.75, 129.05, 128.00, 127.61, 124.80, 123.67, 119.55, 119.53, 116.37, 112.78, 48.22, 21.13, 20.97, 18.94. HRMS (ESI-TOF) m/z: [M + H]^+^ Calcd for C_18_H_17_N_2_ 261.1386; Found 261.1395.

**1-(3-fluorophenyl)-4,9-dihydro-3H-pyrido[3,4-b]indole (6)**

Following the general procedure crude **6a** (250 mg)(which was synthesized *via* general protocol from tryptamine and 3-fluorobenzaldehyde (124 mg, 1 mmol)), with NBS (196 mg, 1.1 mmol) in toluene (10 mL) provided the desired compound **6** in 200 mg (yield 75%) as a white solid. ^1^H NMR (500 MHz; DMSO-d_6_): 11.18 (s, 1H); 7.63-7.57 (m, 3H); 7.53-7.51 (m, 1H) ) 7.44-7.42 (d,*J* = 8.25 Hz, 1H); 7.38-7.7.37 (t, 1H); 7.24-7.20 (m, 1H); 7.09- 7.06 (m, 1H); 3.92-3.89 (t, *J_1_*= 16.45 Hz, *J_2_* = 8.25 Hz, 2H); 2.89- 2.85 (m, 2H). ^13^C NMR (125 MHz; DMSO-d_6_): 163.10, 137.03, 130.60, 130.54, 127.18, 124.76, 124.22, 123.91, 119.68, 119.64, 116.70, 116.53, 114.79, 114.60, 112.80, 43.40, 21.10. HRMS (ESI-TOF) m/z: [M + H]^+^ Calcd for C_17_H_14_FN_2_265.1136; Found 265.1149; IR 2920.22, 1704.91, 1538.20, 1229.07.

**1-(2,4,5-trimethoxyphenyl)-4,9-dihydro-3H-pyrido[3,4-b]indole (7)**

Following the general procedure crude **7a** (300 mg)(which was synthesized *via* general protocol from tryptamine and 2,4,5-methoxybenzaldehyde (197 mg, 1 mmol)), with TFA and NBS (196 mg, 1.1 mmol) in toluene (10 mL) provided the desired compound **7** in 205 mg (yield 61%) as a light brown solid.^1^H NMR (500 MHz; DMSO-d_6_): 10.72 (s, 1H); 7.56-7.55 (d,*J* = 7.55 Hz, 1H); 7.37-7.35 (d, *J* = 8.25 Hz,1H); 7.16-7.13 (m, 1H); 7.04-7.01 (t,*J_1_*= 15.1 Hz, *J_2_* = 8.25 Hz, 1H); 6.93 (s, 1H); 6.82 (s, 1H); 3.88 (s, 5H); 3.72- 3.70 (d,*J* = 6.9 Hz, 6H); 2.87-2.83 (t, *J_1_* = 8.9 Hz, *J_2_* = 8.9 Hz, 2H). ^13^C NMR (125 MHz; DMSO-d_6_): 158.10, 151.72, 150.10, 142.65, 136.55, 128.27, 124.61,123.27, 119.39, 119.14, 113.82, 113.77, 113.53, 98.23, 56.17, 56.11, 56.00, 48.43, 18.89. HRMS (ESI-TOF) m/z: [M + H]^+^Calcd for C_20_H_21_N_2_O_3_337.1547; Found 337.1564;IR. 3349.28, 2836.03, 1580.16, 1435.78, 1277.52

**1-(4-methoxy-3-methylphenyl)-4,9-dihydro-3H-pyrido[3,4-b]indole (8)**

Following the general procedure crude **8a** (288 mg)(which was synthesized *via* general protocol from tryptamine and 3-methyl-4-methoxybenzaldehyde (150 mg, 1 mmol)), with NBS (196 mg, 1.1 mmol) in toluene (10 mL) provided the desired compound **8** in 258 mg (yield 89%) as a colorless gummy solid.. ^1^H NMR (500 MHz; DMSO-d_6_): 11.12 (s, 1H); 7.62-7.60 (t,*J_1_* = 15.1 Hz, *J_2_* = 8.2 Hz, 3H); 7.45-7.43 (d,*J* = 8.25 Hz, 1H); 7.21-7.19 (t,*J_1_* = 15.1 Hz, *J_2_* = 7.55 Hz, 1H); 7.08-7.05 (t,*J_1_* = 14.4 Hz, *J_2_* = 8.2 Hz, 2H); 3.86 (m, 5H); 2.86-2.83 (t,*J_1_* = 15.8 Hz, *J_2_* = 8.2 Hz, 2H); 2.23 (s, 3H). ^13^C NMR (125 MHz; DMSO-d_6_): 158.74, 158.12, 136.90, 130.19, 129.37, 127.64, 127.22, 125.54, 124.80, 123.60, 119.52, 119.49, 116.38, 112.77, 109.96, 55.49, 47.99, 18.96, 16.23. HRMS (ESI-TOF) m/z: [M + H]^+^Calcd for C_19_H_19_N_2_O291.1492 Found 291.1510; IR 2918.44, 1708.58, 1502.94, 1440.37.

**General Procedure for the synthesis of indolo[2,3-a]quinolizidine and indolo[8, 7-b]indolizidine scaffolds** (**9** and **11**)

Appropriate β-aryl amines (0.5 mmol, 1 equiv) and  or γ-oxo esters (0.5 mmol, 1 equiv) were dissolved in CH_3_CN (22 mL) and 10 mol% of trifluoroacetic acid was added to the resulting solution. The reaction mixture was then stirred under argon at 80 °C for 10-16h. Once thin layer chromatography (TLC) confirms the complete consumption of the starting materials, the reaction was cooled to room temperature (rt), quenched with water and was extracted with ethyl acetate. The organic layer was separated, dried over anhydrous sodium sulphate (Na_2_SO_4_) and was evaporated to obtain the desired crude compounds as yellow to colorless solids. The crude solids were purified by flash column chromatography (FCC) using ethyl acetate-hexane as the mobile phase, to provide the final compounds as solid or semi solid, which were characterized by ^1^H-NMR, ^13^C-NMR and QTof-HRMS. Single crystal X-ray structures were obtained for few of them.

*11b-methyl-5,6,11,11b-tetrahydro-1H-indolizino[8,7-b]indol-3(2H)-one* (**9**). Yield: 59 mg, 49%; colorless solid. ^1^H NMR (300 MHz, DMSO-D_6_): * *11.01 (s, 1H), 7.39 and 7.28 (d, *J* = 8 Hz, 2H), 7.05 and 6.95 (t, *J* = 7.2 Hz, 2H), 4.21 (m, 1H), 3.01 (m, 1H), 2.78 (m, 1H), 2.61 (m, 2H), 2.25 (m, 2H), 2.02 (t, *J* = 8 Hz, 1H), 1.48 (s, 3H). ^13^C NMR (125 MHz, DMSO-D_6_) 169.2, 136.3, 133.2, 126.1, 121.3, 118.7, 117.9, 111.2, 104.5, 52.3, 51.9, 49.3, 31.7, 28.9, 22.4, 19.3, 14.1. Q-Tof LCMS(MS ESI): [M + H]^+^calcd for C_15_H_16_N_2_O 241.1335, found 241.1346.

*1, 2, 3, 6, 7, 12b-hexahydroindolo[2,3-a]quinolizin-4(12H)-one* (**11**). Yield: 97 mg, 81%; colorless solid. ^1^H NMR (400 MHz, DMSO-D_6_): *δ* 10.91 (s, 1H), 7.40 and 7.32 (2 X d, *J* = 8 Hz, 2H), 7.06 and 6.97 (2 X t, *J* = 7.2 Hz, 2H), 4.91 and 4.78 (2 X m, 2H), 2.78 (m, 1H), 2.65 (m, 3H), 2.28 (m, 2H), 1.79 (m, 2H), 1.63 (m, 1H). ^13^C NMR (125 MHz, DMSO-D_6_) 168.6, 137.5, 125.8, 121.2, 118.9, 117.9, 109.4, 108.4, 53.8, 31.8, 31.2, 29.3, 21.2, 18.9. Q-Tof LCMS (MS ESI): [M + H]^+^ calcd for C_15_H_16_N_2_O 241.1335, found 241.1376.

**General Procedure for the synthesis of 3, 3´-pyrrolidinylspirooxoindole** (**10a/b**)**.**

To a solution of indolo[2,3-a]quinolizidine and indolo[8, 7-b]indolizine scaffolds (0.5 mmol, 1 equiv) in H_2_O: acetic acid : THF (1:1:1) at 0°C was added NBS (1.2 equiv) in portions. The reaction mixture was stirred at same temperature and after TLC indicates complete consumption of starting material, saturated sodium bicarbonate (NaHCO_3_) solution was added to the reaction mixture and then extracted with EtOAc. The organic layer was washed with brine, dried over anhydrous Na_2_SO_4_ and concentrated under reduced pressure to provide the crude material, which was purified by flash chromatography with EtOAc-Hexane (4:1) as eluent.

*7a'-methyl-2',3',7',7a'-tetrahydrospiro[indoline-3,1'-pyrrolizine]-2,5'(6'H)-dione* (+/-**10a**) and (+/-**10b**). Yield: 94 mg, 74% (combined yield); yellow solid. (+/-) **10a**: ^1^H NMR (400 MHz, DMSO-D_6_): *δ* 10.41 (s, 1H), 7.35 (d, *J* = 7.55 Hz, 1H), 7.21 (t, *J* = 8.25 Hz, 1H), 7.01 (t, *J* = 7.55 Hz, 1H), 6.81 (d, *J* = 7.55 Hz, 1H), 3.65 (m, 1H), 3.17 (m, 1H), 2.61 (m, 2H), 2.25 (m, 1H), 2.18 (m, 1H), 1.79 (m, 1H), 1.59 (m, 1H), 1.39 (s, 3H). ^13^C NMR (75 MHz, DMSO-D_6_) 180.1, 174.8, 142.7, 128.4, 127.6, 125.6, 121.2, 109.4, 71.5, 57.4, 34.6, 33.0, 28.0, 25.6. Q-Tof LCMS (MS ESI): MS ESI): [M + H]^+^ calcd for C_15_H_16_N_2_O_2_ 257.1285, found 257.1290.

(+/-) **10b**. ^1^H NMR (400 MHz, DMSO-D_6_): *δ* 10.45 (s, 1H), 7.21 (t, *J* = 8.25 Hz, 1H), 6.95 (t, *J* = 7.55 Hz, 1H), 6.83 (d, *J* = 7.55 Hz, 1H), 6.65 (d, *J* = 7.55 Hz, 1H), 3.65 (m, 1H), 3.35 (m, 1H), 2.71 (m, 2H), 2.15 (m, 1H), 1.95 (m, 1H), 1.59 (m, 1H), 1.39 (s, 3H), 1.28 (m, 1H). ^13^C NMR (75 MHz, DMSO-D_6_) 176.5, 173.4, 141.4, 132.3, 128.2, 122.8, 121.7, 109.5, 72.7, 57.1, 35.2, 33.1, 27.4, 23.9. Q-Tof LCMS (MS ESI): [M + H]^+^ calcd for C_15_H_16_N_2_O_2_ 257.1285, found 257.1299.

Compound **5**


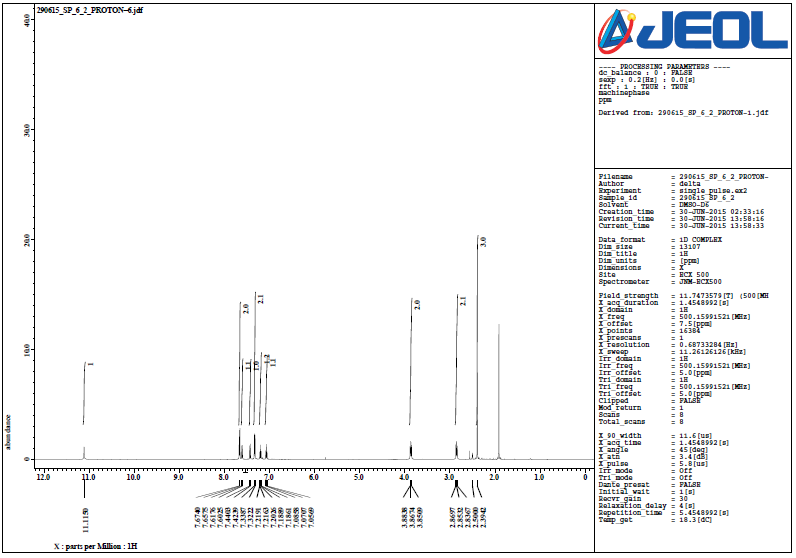


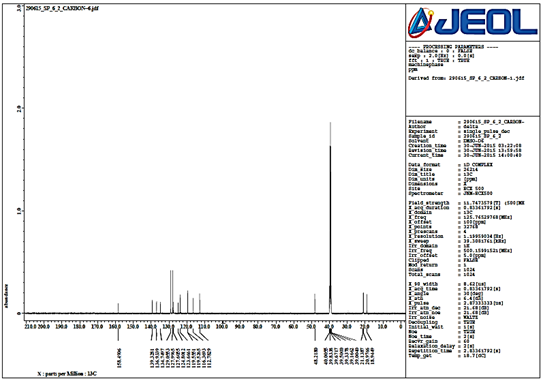


Compound **6**


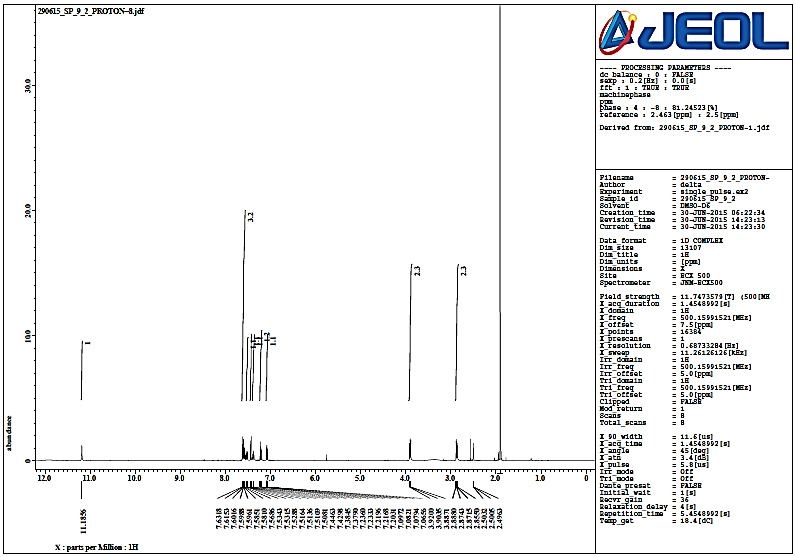


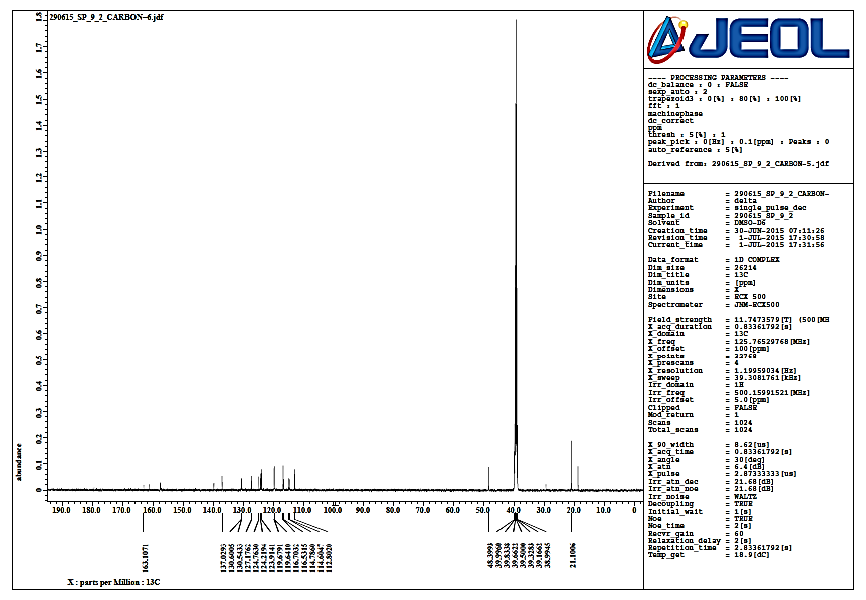


Compound **7**


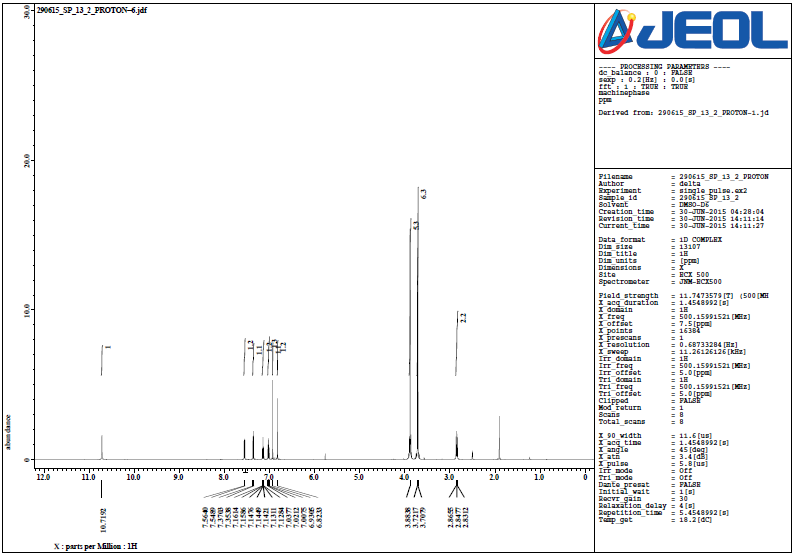


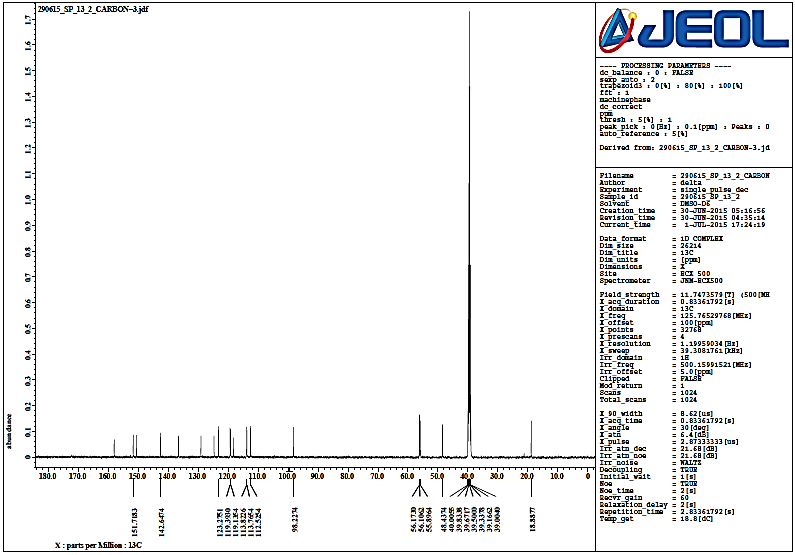


Compound **8**


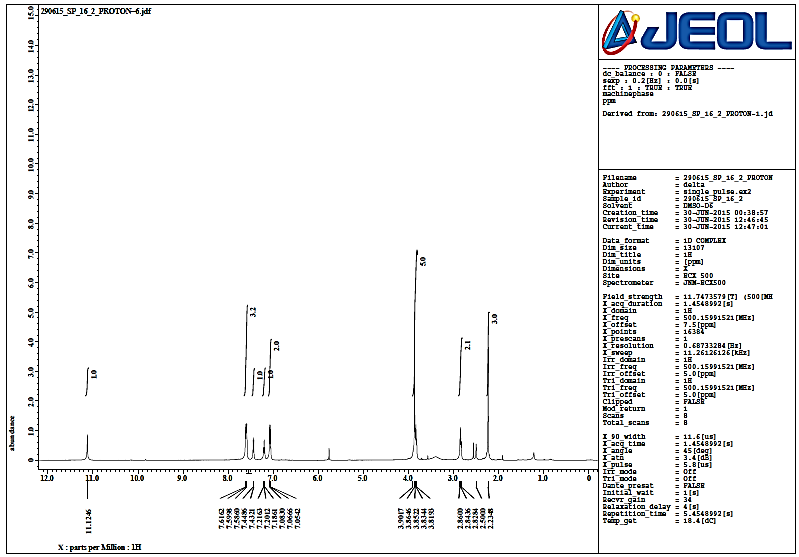


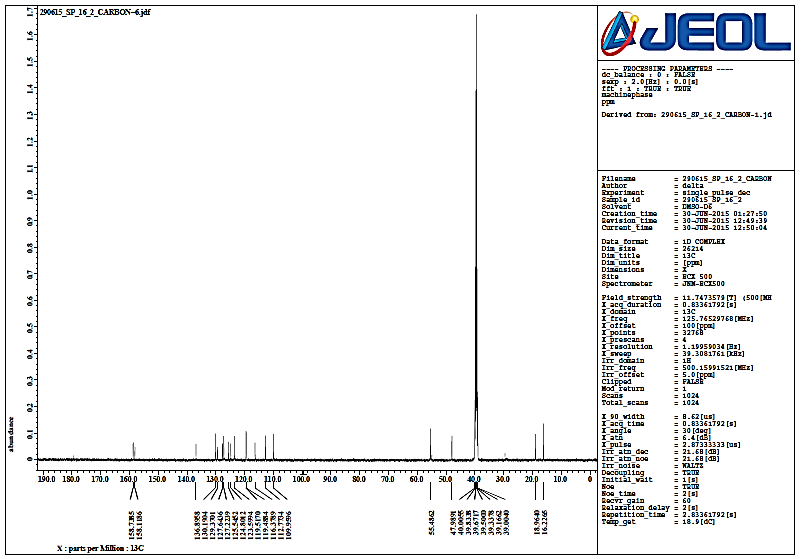


Compound **4**


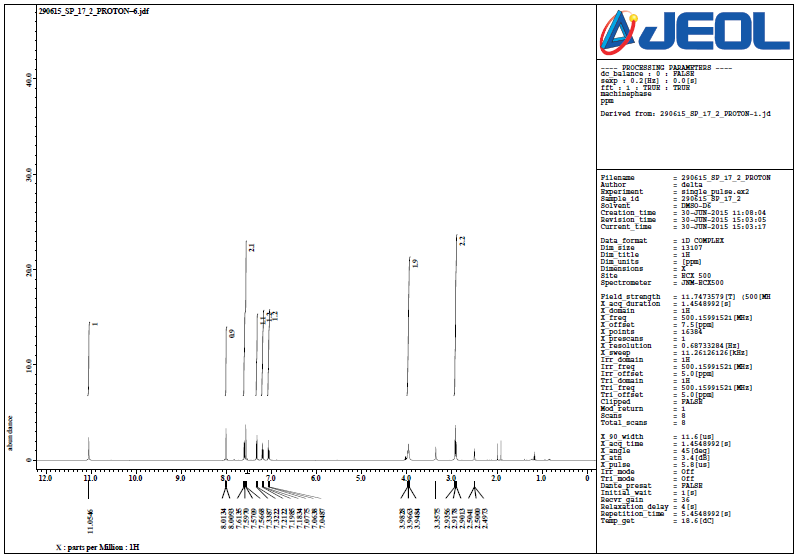


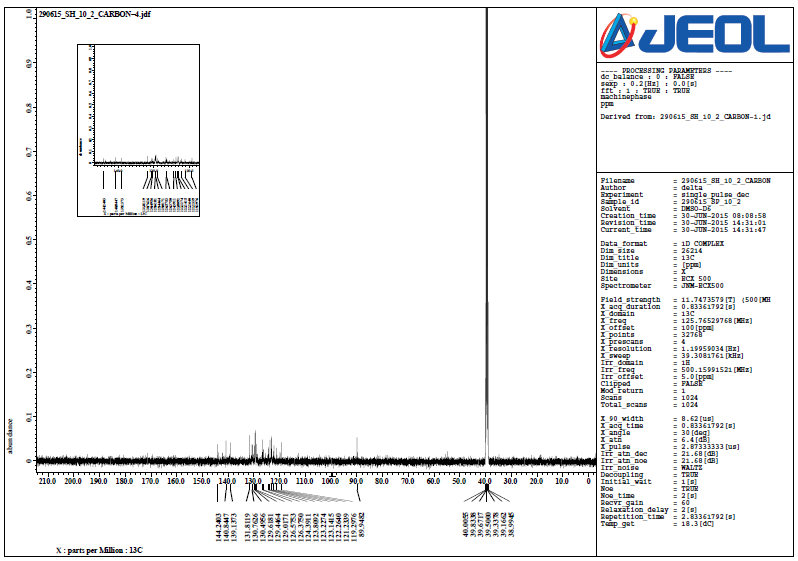


Compound **11**


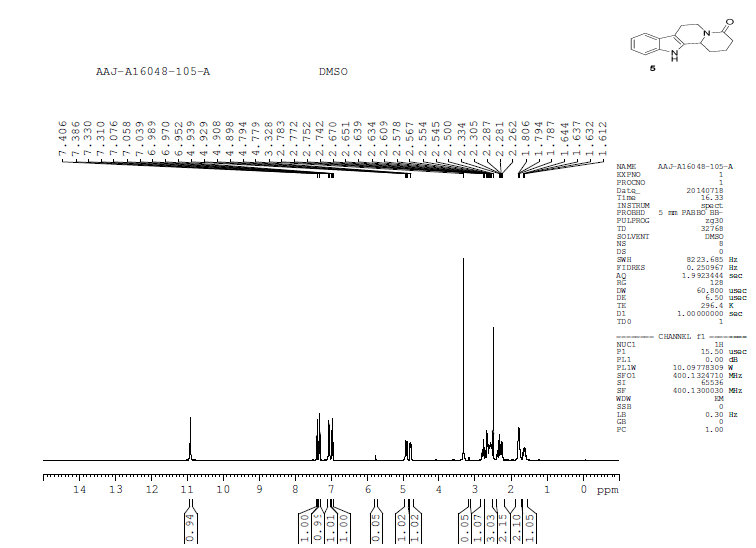


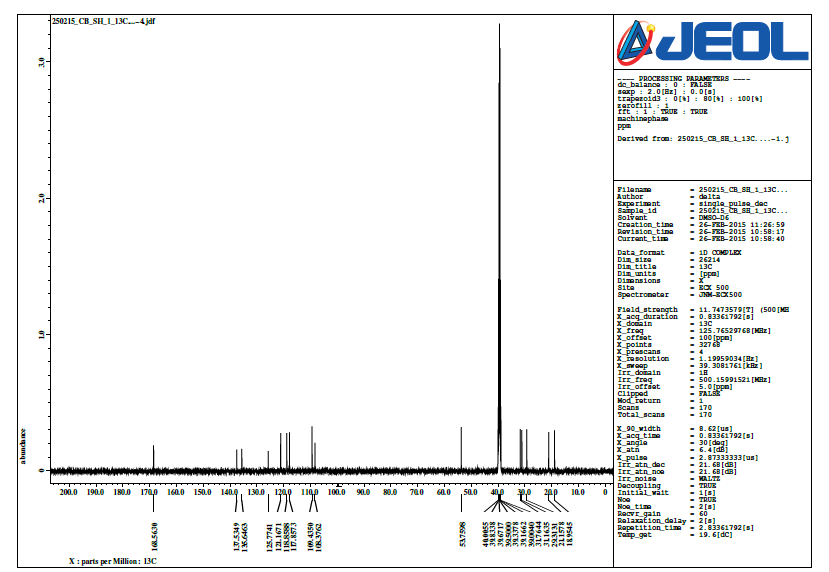


Compound **9**


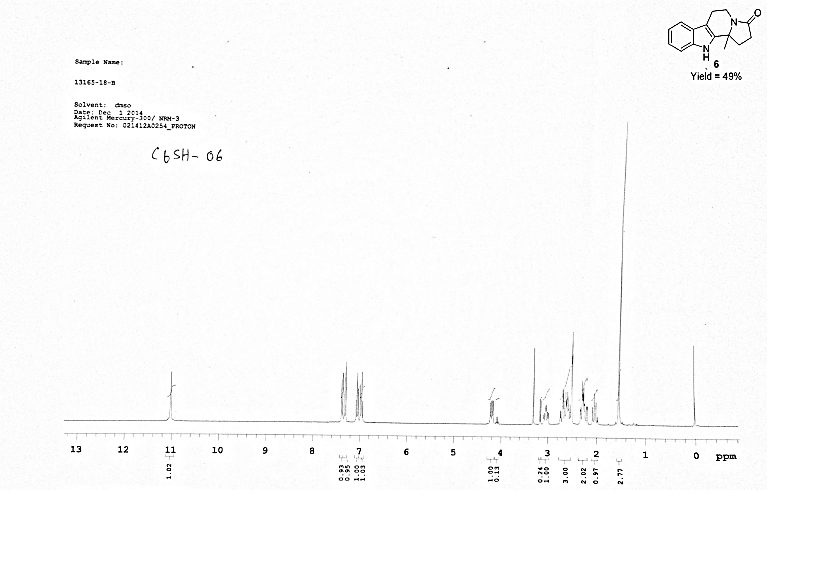


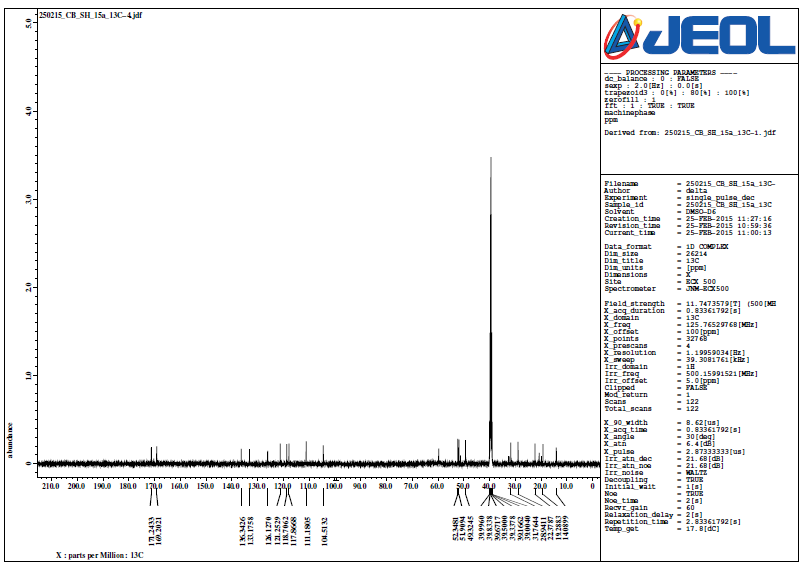


Compound **10a**


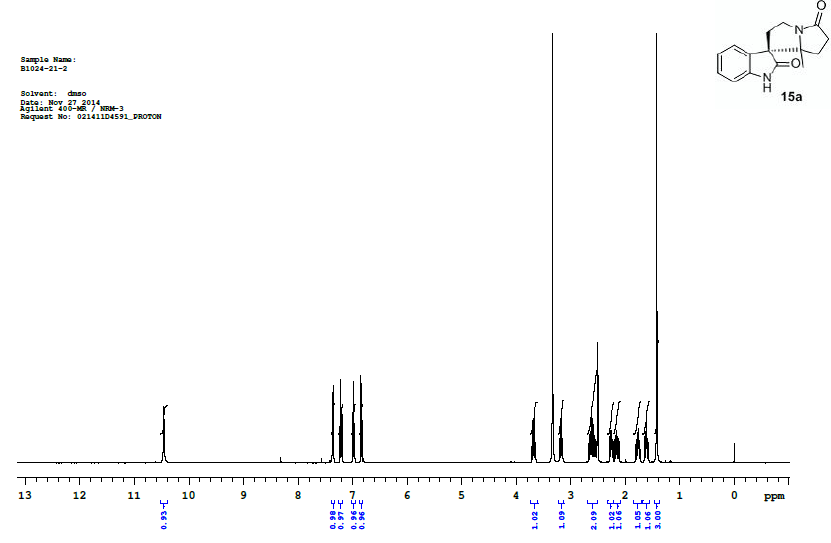


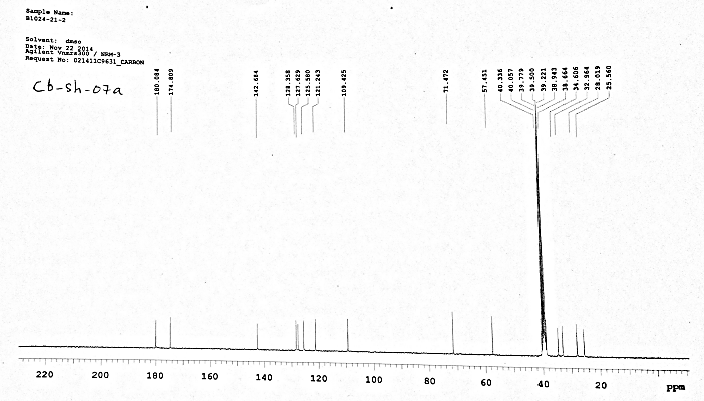


Compound **10b**


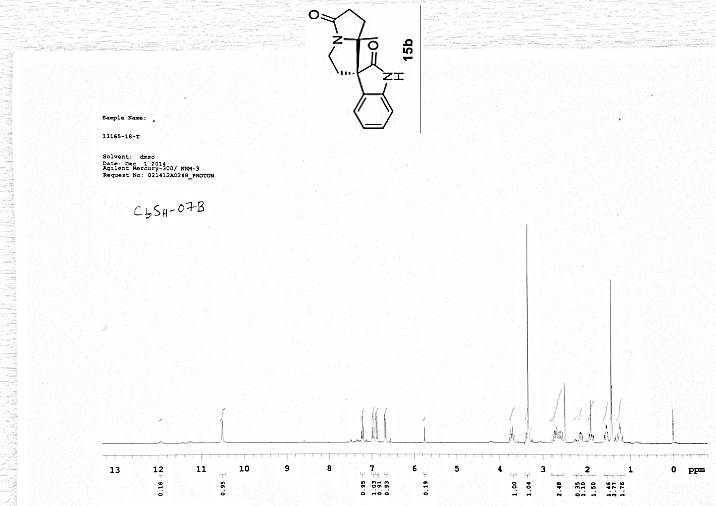


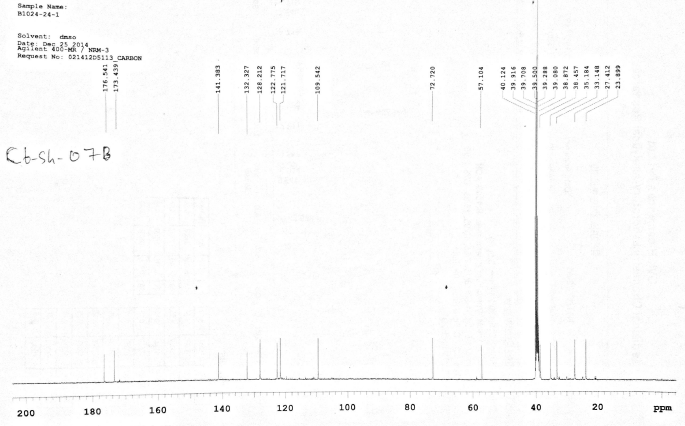

Supplement: Supplementary file 1 — Additional file 1. Supplementary Material. [file 12936_2021_3632_MOESM1_ESM.docx]
